# Physical and mental health impacts of COVID-19 on healthcare workers: a scoping review

**DOI:** 10.1186/s12245-020-00299-5

**Published:** 2020-07-20

**Authors:** Natasha Shaukat, Daniyal Mansoor Ali, Junaid Razzak

**Affiliations:** 1 Department of Community Health Sciences, Aga Khan University, Stadium Road, P.O. Box-3500, Karachi, Pakistan; 2 Centre of Excellence Trauma and Emergencies, Aga Khan University, Karachi, Pakistan; 3 Centre for Global Emergency Care, Department of Emergency Medicine, Johns Hopkins University School of Medicine, Baltimore, USA

**Keywords:** COVID-19, Healthcare workers, Health impacts, Risk factors, Occupational health

## Abstract

**Background:**

Coronavirus disease (COVID-19) pandemic has spread to 198 countries, with approximately 2.4 million confirmed cases and 150,000 deaths globally as of April 18. Frontline healthcare workers (HCWs) face a substantially higher risk of infection and death due to excessive COVID-19 exposure. This review aimed at summarizing the evidence of the physical and mental health impacts of COVID-19 pandemic on health-care workers (HCWs).

**Methods:**

We used the Arksey O’Malley framework to conduct a scoping review. A systematic literature search was conducted using two databases: PubMed and Google Scholar. We found 154 studies, and out of which 10 met our criteria. We collected information on the date of publication, first author’s country, the title of the article, study design, study population, intervention and outcome, and key findings, and divided all research articles into two domains: physical and mental health impact.

**Results:**

We reviewed a total of 154 articles from PubMed (126) and Google Scholar (28), of which 58 were found to be duplicate articles and were excluded. Of the remaining 96 articles, 82 were excluded after screening for eligibility, and 4 articles did not have available full texts. Ten full-text articles were reviewed and included in this study.

Our findings identified the following risk factors for COVID-19-related health impact: working in a high-risk department, diagnosed family member, inadequate hand hygiene, suboptimal hand hygiene before and after contact with patients, improper PPE use, close contact with patients (≥ 12 times/day), long daily contact hours (≥ 15 h), and unprotected exposure. The most common symptoms identified amongst HCWs were fever (85%), cough (70%), and weakness (70%). Prolonged PPE usage led to cutaneous manifestations and skin damage (97%), with the nasal bridge (83%) most commonly affected site. HCWs experienced high levels of depression, anxiety, insomnia, and distress. Female HCWs and nurses were disproportionately affected.

**Conclusion:**

The frontline healthcare workers are at risk of physical and mental consequences directly as the result of providing care to patients with COVID-19. Even though there are few intervention studies, early data suggest implementation strategies to reduce the chances of infections, shorter shift lengths, and mechanisms for mental health support could reduce the morbidity and mortality amongst HCWs.

## Background

Coronavirus disease 2019 (COVID-19) was first identified in Wuhan City in December 2019, after which, the disease spread throughout Hubei Province and other parts of China [1, 2]. After causing significant morbidity and mortality in China, by February 2020, COVID-19 had spread to numerous other countries, including the USA, Italy, Spain, Germany, France, and Iran [3–5]. As of April 18, COVID-19 has spread to 198 countries, infecting 2.4 million people and causing 150,000 deaths across the world and is therefore considered a global pandemic [6–8].

Healthcare workers (HCWs) are amongst the high-risk group to acquire this infection [9–11]. China reported infection in 3387 HCWs, while 22 HCWs (0.6%) died due to the illness [9, 12]. Similarly, Italy (20%), Spain (14%), and France (over 50 deaths amongst HCWs) reported high rates of HCW infection [10, 13, 14].

Given the high burden, there is a growing demand and focus on protecting HCWs across the world through provision of personal protective equipment (PPE), training, addressing fatigue, and countering the psychosocial consequences [15–21].

The literature on the health consequences of HCWs providing care to COVID-19 patients is proliferating, and no review is available to guide practitioners and leaders on the efficacy of various interventions. This scoping review aims to summarize the evidence of the physical and mental health impacts of COVID-19 pandemic on healthcare workers.

## Methods

### Study design

We used the methodological framework by Arksey and O’Malley to conduct the scoping review [22]. The five steps followed were identifying a clear research question and objective; identifying relevant articles; selection of articles, data extraction; and charting of data, organizing, summarizing, analyzing, and reporting of data [22]. The primary research question guiding this review is “What are the physical and mental health effects of managing patients with COVID-19 on the frontline health-care workers?”

### Literature search strategies

We searched PubMed or Medical Literature Analysis and Retrieval System Online (MEDLINE), and Google Scholar for relevant articles from January to March 2020. Medical subject headings (MeSH) were searched using Boolean operators “*OR/AND*”. The search terms were: (“2019-nCoV” OR “coronavirus” OR “COVID-19” OR “nCoV)” AND (“health-care workers”) AND (“health impacts” OR “physical health” OR “mental health”).

### Eligibility criteria

We included studies assessing the impact of COVID-19 on the health of HCWs and were published in the English language and published from January to March 2020. Healthcare workers included all clinical staff, including doctors, nurses, paramedics, and technicians. Editorials, commentaries, and non-English articles were excluded.

### Identification and selection of studies

**Fig. 1:**
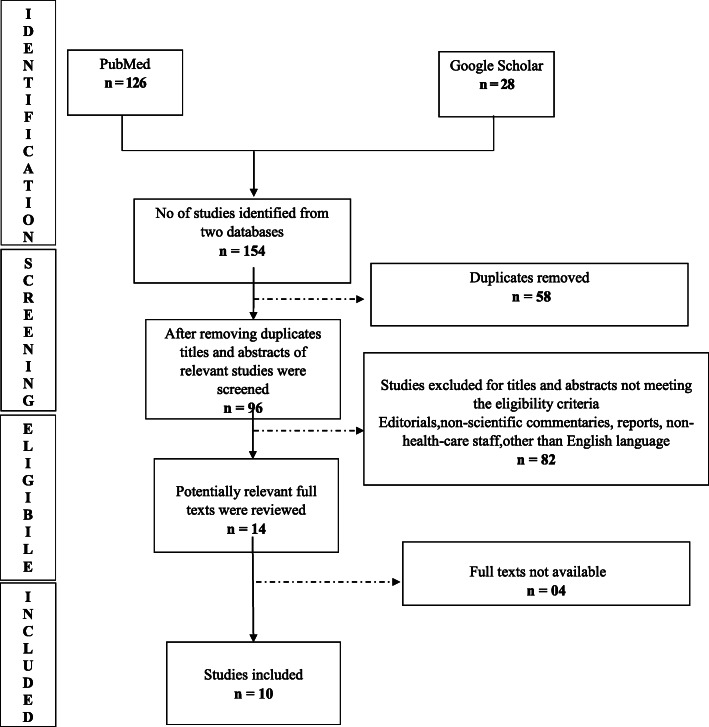
PRISMA flow diagram for database search of studies

Two researchers (NS and DMA) independently searched through the literature. The two sets of literature were then compared, and duplicate articles were removed. Figure 1 presents a Preferred Reporting Items for Systematic Reviews and Meta-Analyses (PRISMA) flow diagram showing the process of searching and selecting the research articles.

### Data extraction from included studies

After the selection of the articles, we extracted and recorded data in a data extraction form in an Excel spreadsheet. The domains in the data extraction form were date of publication, the title of the article, name of the journal, study design, study setting and population, intervention, and outcome reported and key findings.

### Summarizing the findings

We summarized our findings into the following research domains: mental health impacts and physical health impacts.

## Results

### Studies’ characteristics

A total of 154 articles were retrieved from PubMed (126 articles) and Google Scholar (28 articles). Fifty-eight duplicate articles were excluded. Out of the remaining 96 articles, 82 articles were either not related to the impact of COVID-19 on healthcare workers, were editorials or commentaries, or were written in a language other than English, and no English translation was available and therefore were excluded. Among the remaining 14 articles, 4 articles did not have available full texts. Ten full-text articles were reviewed and included in this study. Out of the ten studies included, two were written in Chinese but had English translations available.

### Research domains

Among the 10 studies included in this review, 5 studies assessed mental health impacts, and 5 studies assessed the physical health impacts of COVID-19 on healthcare workers. The methodological characteristics of these studies are summarized in Table 1. Six were cross-sectional, two were interventions, one was a retrospective cohort, and one was a case series. The study population comprised both male and female, including frontline physicians, nurses, and specialist staff. Most of the studies (90%) were from scholars in the Peoples Republic of China (PRC), while one was from scholars based in Singapore.

**Table 1:** Methodological characteristics of COVID-19 research articles

Research domain	Study design *n* (%)	Study population *n* (%)
Mental health impact	Cross-sectional study 3 (30%) Interventional study 2 (20%)	Healthcare workers (nurses and physicians) 4143
Physical health impact	Cross-sectional study 3 (30%) Retrospective cohort study 1 (10%) Case series 1 (10%)	Healthcare workers (nurses, frontline physicians and ICU medical staff) 1267

The findings related to mental health and physical health impact of COVID-19 on healthcare workers from the included articles are summarized in Table 2.

**Table 2:** Summarized findings of included COVID-19 research articles

Author, year, country	Study title	Study design and population	Intervention/outcome	Key findings
Mental health impacts				
Xiao H et al. 2020, China	The Effects of Social Support on Sleep Quality of Medical Staff Treating Patients with Coronavirus Disease 2019 (COVID-19) in January and February 2020 in China	Cross-sectional study *N* = 180	Anxiety, self-efficacy, stress, sleep quality, social support	High levels of anxiety, stress, and self-efficacy were associated with sleep quality and social support
Huang JZ et al. 2020, China	Mental health survey of 230 medical staff in a tertiary infectious disease hospital for COVID-19	Cross-sectional study *N* = 246	Mental health status (anxiety and post-traumatic stress disorder)	Overall anxiety (23.04%) Severe anxiety (2.17%) Moderate anxiety (4.78%) Mild anxiety (16.09%) Anxiety in females higher than males (25.67% vs. 11.63%) Anxiety in nurses higher than doctors (26.88% vs. 14.29%) Stress disorder (27.39%)
Chen Q et al. 2020, China	Mental health care for medical staff in China during the COVID-19 outbreak	Correspondence *N* = 1230	**Intervention:** place of rest, food and daily supply for staff, video recording of the daily routine of staff, pre-job training to deal with psychological problems in patients, PPE, leisure activities and training to relax, psychological counselors **Outcome:** irritability, unwillingness to rest, psychological distress before and after intervention	The learning from psychological interventions is expected to help the Chinese government and other parts of the world to better respond to future unexpected infectious disease outbreaks
Kang L et al. 2020, Wuhan, China	The mental health of medical workers in Wuhan, China dealing with the 2019 novel coronavirus	Correspondence *N* = 1230	**Intervention:** Built psychological intervention medical team, hotline, various group activities to release stress **Outcome**: change in stress, anxiety, depressive symptoms, insomnia denial, anger and fear before and after intervention	This approach provides multifaceted psychological protection of the mental health of medical workers.
Jianbo Lai et al. 2020, China	Factors associated with Mental Health outcomes Among Health Care Workers Exposed to Coronavirus Disease 2019	Cross-sectional study *N* = 1257, 34 hospitals	Depression, anxiety, insomnia, and distress	Depression (50.4%) Anxiety (44.6%) Insomnia (34.0%) Distress (71.5%) More psychological burden among nurses, women, those in Wuhan, and frontline healthcare workers
Physical health impacts				
Ran L et al. 2020, Wuhan, China	Risk factors of Healthcare Workers with Corona Virus Disease 2019: A Retrospective Cohort Study in a Designated Hospital of Wuhan in China	Retrospective cohort study *N* = 83	Sociodemographic characteristics, time to symptomatic progression, contact history, medical practice, hand hygiene, and PPE	28 HCWs were diagnosed with COVID-19 Diagnosed family member (*p* < 0.01), unqualified hand-washing (*p* < 0.05), suboptimal hand hygiene before (*p* < 0.01), and after (*p* < 0.01) contact with patients Improper PPE (*p* < 0.05) were associated with increased risk of infection
Liu M et al., 2020, China	Clinical characteristics of 30 medical workers infected with new coronavirus pneumonia	Cross-sectional study *N* = 30	Clinical characteristics of medical staff with novel coronavirus pneumonia	Total of 30 cases, 26 mild cases, and 4 severe cases Cough (83.33%) Fever (76.67%) were the most common symptoms
Lan J et al. 2020, Hubei, China	Skin damage among health-care workers managing coronavirus disease-2019	Cross-sectional study *N* = 700	Cutaneous complications related to preventative measures among health-care workers treating patients with COVID-19	Prevalence of skin damage: 97% Nasal bridge most common site: 83.1% Dryness/tightness: 70.3%
Kangqi Ng et al. 2020, Singapore	COVID-19 and the Risk to Health Care Workers: A Case Report	Case report *N* = 41	The rate of infection in 41 health-care workers exposed to the patient with COVID-19 during aerosol-generating procedure	None of the healthcare workers got infected by COVID-19 85% of healthcare workers were wearing surgical masks while 15% were wearing N-95 during aerosol-generating procedure Surgical masks, hand hygiene, and other standard procedures were sufficient to protect against infection
Vincent C.C. Cheng et al. 2020, Hongkong	Escalating infection control response to the rapidly evolving epidemiology of the Coronavirus disease 2019 (COVID-19) due to SARS-CoV-2 in Hong Kong	Cross-sectional study *N* = 413	Contact tracing of HCWs with unprotected exposure	2.7% (11/413) HCWs had unprotected exposure, none of them were infected

### Mental health impacts

Five articles discussed mental health impact on healthcare providers. In one study, out of 230 healthcare workers who responded to the mental health assessment scales, 53 (23.04%) had psychosocial problems. Among these 53 medical staff, more females (48 (90.57%)) than males (5 (9.43%)), and more nurses (43 (81.13%)) than physicians (10 (18.9%)) suffered from mental health issues due to the infectious outbreak [23]. The psychological impact on healthcare workers included the following conditions: overall anxiety (23–44%), severe anxiety (2.17%), moderate anxiety (4.78%), mild anxiety (16.09%), stress disorder (27.4–71%), depression (50.4%), and insomnia (34.0%) [23, 24]. Anxiety in females was higher than in males (25.67% vs. 11.63%), nurses higher than doctors (26.88% vs. 14.29%) [23]. Frontline healthcare workers engaged in direct COVID-19 patient care were at higher risk of depression (OR 1.52; 95% CI 1.11–2.09), anxiety (OR,1.57; 95% CI 1.22–2.02), insomnia (OR 2.97; 95% CI 1.92–4.60), and distress (OR 1.60; 95% CI 1.25–2.04) [24].

The tools used in these studies included Self-Rating Anxiety Scale [23, 25], Generalized Anxiety Disorder Scale [24], General Self-Efficacy Scale [25], Stanford Acute Stress Reaction Questionnaire [25], Pittsburgh Sleep Quality Index [25], Insomnia Severity Index [24], Social Support Rate Scale [25], Post-Traumatic Stress Disorder Self-Rating Scale [23], and Impact of Event Scale [24].

### Physical health impacts

#### COVID-19 infection transmission and mortality among healthcare providers

Early studies from the Peoples Republic of China (PRC) demonstrate that HCWs are more susceptible to COVID-19. Studies amongst HCWs in PRC showed that COVID-19 risk was linked with working in high-risk department such as infectious disease and pulmonology (RR = 2.13, 95% CI 1.45–3.95), diagnosed family member (RR = 2.76, 95% CI 2.02–3.77), inadequate hand hygiene (RR = 2.64, 95% CI 1.04–6.71), suboptimal hand hygiene before and after contact with patients (RR = 2.43, 95% CI 1.34–4.39), improper PPE (RR = 2.82, 95% CI 1.11–7.18), close contact with patients (12 times/day), long daily contact hours (≥ 15 h), and unprotected exposure. Common symptoms were fever (85%), cough (80%), weakness (70%), chest distress (7%), hemoptysis (7%), headache (7%), and diarrhea (7%) [17, 26, 27]. Similarly, another study showed that COVID-19 infected 30 medical staff, including 20 doctors and 8 nurses in a hospital. Of these, 26 had mild, and 4 had a severe infection, and all of them had exposure to the virus [27]. A case series from Singapore recorded outcomes of 41 HCWs who were exposed to a patient with COVID-19 pneumonia before diagnosis of COVID in this patient. None of the 41 HCWs developed COVID-19. All the HCWs were wearing surgical and N-95 masks at the time of exposure [28].

#### Cutaneous manifestations

Prevention against the viral illness meant that healthcare workers had to wear personal protective equipment (PPE) for a prolonged period. A cross-sectional study demonstrated skin damage in 97% of the medical staff, with the nasal bridge (83.1%), being the most commonly affected site. The most common presenting symptom was dryness or tightness and desquamation (70.3%), and these manifestations were associated with more than 6 h of continuous PPE use and more than 10 times/day hand hygiene [29].

## Discussion

This review collates evidence on the health impacts of COVID-19 on HCWs. Our findings suggest HCWs are susceptible to various health consequences due to the COVID-19 pandemic. For those with COVID-19 infections, the most common symptoms were fever and cough, which were similar to those seen in the community. Several risk factors were identified; long duty hours, working in the high-risk department, lack of PPE, diagnosed family member, unqualified hand-washing, and improper infection control. Furthermore, prolonged PPE usage led to skin damage, with the nasal bridge being the most common site. Battling COVID-19 on the frontline makes HCWs vulnerable to psychological distress. Finding shows high levels of depression, stress, anxiety, distress, anger, fear, insomnia, and post-traumatic stress disorder in the HCWs. Females and nurses were disproportionately affected more from mental health consequences. Frontline female nurses work in close contact with patients for longer working hours, which may result in fatigue, stress, and anxiety. However, this finding warrants for further research to better prepare for the future.

Worldwide, COVID-19 has affected large numbers of frontline HCWs. As of March 2020, COVID-19 has infected more than 3000 HCWs in China only [30]. A similar situation was witnessed in previous outbreaks of Ebola virus disease (EVD), Middle East respiratory syndrome (MERS), and severe acute respiratory syndrome (SARS) [31–35]. Figures from Sierra Leone, Liberia, and Guinea showed approximately 6–8% of Ebola infection amongst the HCWs [35], SARS infected approximately 1000 HCWs, and 1.4% deaths occurred in China only [36]. Early COVID-19 studies indicate a worrisome situation of morbidity and mortality [16, 20]. The fact that healthcare workers are at increased risk of infection by COVID-19 will further exacerbate the existing shortage of skilled workforce, as most health systems and EDs are running at their full capacities [18, 20, 30, 37].

During outbreaks, the HCWs experience considerable stress. In a Chinese study during the Ebola outbreak, HCWs reported extreme somatization, depression, anxiety, and obsession-compulsion [38]. During the MERS outbreak, a Saudi study reported almost two-third of HCWs felt at risk of getting infected with MERS CoV and felt unsafe at work [39]. These findings are consistent with previous SARS situations in which HCWs reported high levels of fear of contagion and infecting family members, emotional disturbance, uncertainty, and stigmatization [40, 41]. Risk factors for mental health include overwhelming situations, social disruption of daily life, feeling vulnerable, at risk of getting infected, fear of transmitting the disease to families, and loved ones [11]. Previous outbreaks showed that HCWs suffer significant stress, and a similar outcome is expected in COVID-19.

One of the major challenges faced in controlling this pandemic is the extreme shortage of PPEs [18]. A highly infectious pandemic challenges already compromised health systems with resultant shortages in supplies and PPEs. For instance, during the Ebola outbreak, many countries faced PPE shortages [35, 42]. In a pandemic, ensuring emergency medical supplies is pertinent to national public health emergency response systems [18]. Therefore, it is pertinent to establish an emergency reserve medical supplies program to ensure the provision of supplies based on needs, type, quality, and quantity.

Pandemics exert significant psychological impacts on HCWs, highlighting the need for appropriate psychological support, interventions, and staff support measures. COVID-19-specific psychological interventions for medical staff in China included psychological intervention support teams, psychological counselling, availability of helpline, establishment of shift systems in hospitals, online platforms for medical assistance, incentives, providing adequate breaks and time offs, providing a place to rest and sleep, leisure activities such as yoga, meditation and exercise, and motivational sessions [15, 16]. Protecting the well-being of HCWs, through appropriate measures is a crucial tool in national emergency public health response to fighting the outbreaks. If timely measures are not taken, although the disease will subside eventually, a new surge of patients suffering from psychological morbidity will emerge.

### Strengths and limitations of the study

The scoping review applied systematic and vigorous search strategy as per the study objective. The study presents a summary the recent scientific evidence and could strengthen the response for the current and future outbreaks. Given the rapidity of the pandemic, studies present here have a relatively short follow-up period. Also, our review included only studies published in the English language and may have missed findings published in other languages. The incidence of health impacts of COVID-19 on HCWs are not documented due to methodological limitations of studies, especially difficulty in finding the actual denominator data. Lastly, interventional studies are relatively scarce.

## Conclusion

HCWs are at risk for developing physical and mental health consequences due to their role in providing care to patients with COVID-19. Implementation of the following strategies may help reduce the burden of health consequences: the adequate provision and training on the use of personal protective equipment, strict infection control practices, shorter shift length, and provision of mental health and support services.

## Data Availability

The data that support the findings of this study are available from the corresponding author upon reasonable request.

## Abbreviations

COVID-19: Corona virus disease
HCWs: Healthcare workers
PPE: Personal protective equipment
PRISMA: Preferred Reporting Items for Systematic Reviews and Meta-Analyses
EVD: Ebola virus disease
MERS: Middle East respiratory syndrome
SARS: Severe acute respiratory syndrome
